# Design of Quad-Band Terahertz Metamaterial Absorber Using a Perforated Rectangular Resonator for Sensing Applications

**DOI:** 10.1186/s11671-018-2567-5

**Published:** 2018-05-08

**Authors:** Qin Xie, Guangxi Dong, Ben-Xin Wang, Wei-Qing Huang

**Affiliations:** 1 School of Science, Jiangnan University, Wuxi, 214122 China; 2 School of Physics and Electronics, Hunan University, Changsha, 410082 China

**Keywords:** Metamaterial, Quad-band absorption, Terahertz, Sensing

## Abstract

Quad-band terahertz absorber with single-sized metamaterial design formed by a perforated rectangular resonator on a gold substrate with a dielectric gap in between is investigated. The designed metamaterial structure enables four absorption peaks, of which the first three peaks have large absorption coefficient while the last peak possesses a high *Q* (quality factor) value of 98.33. The underlying physical mechanisms of these peaks are explored; it is found that their near-field distributions are different. Moreover, the figure of merit (FOM) of the last absorption peak can reach 101.67, which is much higher than that of the first three absorption modes and even absorption bands of other works operated in the terahertz frequency. The designed device with multiple-band absorption and high FOM could provide numerous potential applications in terahertz technology-related fields.

## Background

Metamaterials with sub- or deep sub-wavelength structure size have received more and more attention because they have been proved to show exotic electromagnetic (EM) properties [1–3] that cannot be directly obtained under the natural condition. In addition to these fascinating effects, metamaterials have also a wide variety of applications in functional devices [4–10]. Metamaterial absorbers, as the special branch of the metamaterial devices, have evoked great interest of researchers because they can be used to achieve large light absorption [6, 11–38].

In the year of 2008, a research group from Boston College first designed the metamaterial absorber at the microwave region by making full use of the dissipation losses of the sandwich structure composed of electric ring resonator, lossy dielectric layer, and the metallic cut wire [6]. Thereafter, various kinds of investigations have been proved based on different shapes or sizes of metallic resonators. For example, Yao et al. presented a miniaturized metamaterial absorber by using a folded-line structure [17]. Cross-shaped terahertz absorber was demonstrated in Ref. [18]. Unfortunately, these demonstrated metamaterial absorbers are limited to the single-band absorption, which can greatly restrict their practical applications. To resolve the issue of the single-band absorption, the design and development of the multiple-band and even broadband light absorbers are necessary.

Results demonstrate that the blending of multiple resonators to form coplanar or layered structures can have the ability to achieve the perfect absorption in multiple frequency bands (i.e., the multiple-band absorption) [22–38]. For example, coplanar structures consisted of several different sizes of closed-ring resonators [22–27], square patches [28, 29], and electric ring resonators [30–33] were presented to realize dual-band and triple-band absorption. Layered structure designs were suggested to also obtain the multiple-band absorption devices [34–38]. In these suggestions, each metallic resonator has only single absorption mode, and therefore, the design of the multiple-band absorption devices requires at least as many resonators as absorption peaks. In Refs. [22–38], we clearly found that the dual-band, triple-band, and even quad-band metamaterial absorbers indeed need at least two, three, and four metallic resonators in a unit cell, respectively. That is to say, previous studies are mainly focused on how to achieve multiple-band absorption by utilizing multiple different sizes of resonators, few of them are investigated whether the single-sized resonator has the ability to exhibit multiple-band absorption responses.

In this paper, we demonstrate that single-sized metallic resonator enables quad-band absorption, which is different from previous design concepts that several resonators with different sizes are needed. The design of quad-band light absorber is composed of a perforated rectangular resonator on a gold mirror with a lossy dielectric layer in between. Numerical results clearly indicate that the designed metamaterial structure possesses four narrow-band absorption peaks, of which the first three peaks have strong absorption of 97.80% on average while the fourth peak has *Q* value of 98.33. With the aid of the near-field distributions, the underlying physical pictures of the quad-band absorption are analyzed. The sensing performance of the suggested light absorption device is also discussed; results prove that the sensing sensitivity (*S*) of the device, in particular of the *S* of the fourth absorption peak, can reach 3.05 THz per refraction index; and the figure of merit (FOM; the definition of the FOM is sensing sensitivity *S* divided by its absorption bandwidth [44, 45]) of this mode can be up to 101.67. The large *S* and high FOM of the designed light absorption device are promising in sensor-related fields.

## Methods

**Fig. 1:**
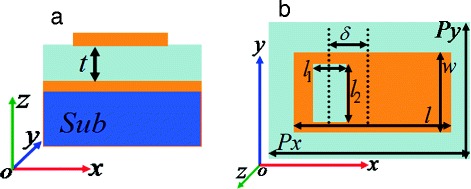
**a** and **b** are respectively the side- and top view of the presented quad-band terahertz metamaterial absorber

Figure 1a shows the side view of the designed quad-band light absorber, it is composed of a perforated rectangular resonator (see Fig. 1b), and a metallic board and a lossy dielectric layer separated them. The metallic layers of the light absorber are made of 0.4 μm gold, and its conductivity is *σ =* 4.09 × 10^7^ S/m. The lossy dielectric-separated layer has the thickness of *t* = 9 μm and the lossy dielectric constant of 3(1 + *i*0.05), and this kind of lossy dielectric material is widely used in the field of metamaterials [46]. The top view of the perforated rectangular resonator is depicted in Fig. 1b, and its geometrical parameters are as follows: the length and width of the rectangular resonator are *l* = 80 μm and *w* = 40 μm, respectively. The length and width of the perforated air hole are *l*_1_ = 25 μm and *l*_2_ = 35 μm, respectively. The deviation value of the perforated air hole is *δ* = 18 μm. The periods in *P*x and *P*y are respectively 100 and 60 μm.

Here, we would like to briefly introduce the design rules of the metasurface, i.e., the single-sized perforated rectangular resonator. In general, the traditional single-sized metallic resonator (for example, closed-ring resonator, square patch, and rectangular resonator) has only one resonance absorption peak, and the design of the multiple-band light absorption devices requires at least as many resonators as absorption peaks. As given and reported in Refs. [22–38], the dual-band, triple-band, and even quad-band light absorption devices indeed need at least two, three, and four metallic resonators in a unit cell, respectively. In other words, previous works are mainly concentrated on how to achieve multiple-band light absorption devices using multiple different sizes (or shapes) of the traditional metallic resonators, few of them are investigated whether the single-sized resonator with the slight structure deformation has the ability to achieve multiple-band absorption. Here, we try to obtain the multiple-band absorption by introducing the breach (i.e., the air hole) on the traditional rectangular metallic resonator. It is foreseeable that the introduction of the air hole on the traditional rectangular resonator can break the symmetry of the original rectangular metallic resonator and can break the original near-field distributions (or the rearrangement of the near-field distributions in the perforated rectangular resonator), thus introducing (or generating) some new resonance absorption modes. As mentioned in Fig. 4, the introduction of the breach (or the air hole) on the traditional rectangular resonator can indeed rearrange the near-field distributions, resulting in some new resonance absorption peaks. Therefore, we believe that the slight structure deformation of the traditional metallic resonator is an effective way to achieve the multiple-band absorption; this kind of design method is bound to have obvious advantages compared to the previous design approaches using multiple different-sized resonators [22–38]. Additionally, for the metamaterial absorber, its 100% absorption can be mainly derived from two aspects, the ohmic loss in metallic layers and the absorption in the dielectric slab using the lossy dielectric. In the frequency bands of terahertz and microwave [6, 18, 23–25, 39, 50], the ohmic loss in metallic layers is usually smaller than the absorption in the dielectric layer. That is to say, it is impossible to merely use the ohmic loss to achieve the 100% absorption. Therefore, it is usually necessary to use the lossy dielectric as the dielectric slab of the metamaterial absorbers [22–37].

The quad-band metamaterial absorber is simulated by employing the commercial software, FDTD Solutions, which is based on the finite difference time domain method. In the calculation, a plane electromagnetic wave with the electric field along the direction of *x*-axis is used as the light source, which is perpendicularly irradiated to the resonance structure (from the perforated resonator to the lossy dielectric layer and the gold mirror) along the direction of the *z*-axis (see Fig. 1). The light source has the frequency range of (0.2~3.0) THz. To ensure the accuracy of calculation results, the size of the light source should be slightly larger than that of the repeated period of the structure, at the same time enough simulation times and the suitable boundaries (periodic boundaries in directions of *x*- and *y*-axis and perfectly matched layers in direction of *z*-axis) should be utilized.

## Results and Discussion

**Fig. 2:**
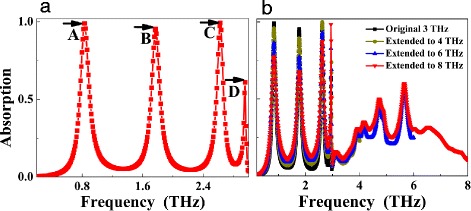
**a** is the absorption performance of the presented quad-band light absorber. **b** shows the dependence of the absorption performance on the extended frequency ranges

Figure 2a gives the absorption performance the presented quad-band terahertz metamaterial absorber. As revealed, the suggested simple-sized structure can have four peaks, the frequencies of them are respectively 0.84 THz in point *A*, 1.77 THz in point *B*, 2.63 THz in point *C*, and 2.95 THz in point *D*. The first three frequency points (*A*, *B*, and *C*) have the large average absorption rates of 97.80%, and the absorption of the frequency point *D* is about 60.86%. The bandwidths (full width at half maximum, abbreviated as FWHM) of the frequency points *A*, *B*, *C*, and *D* are 0.13, 0.13, 0.10, and 0.03 THz, respectively. In general, the *Q* (or quality factor, and the definition of the *Q* is the resonance frequency point divided by its bandwidth) value is a very important indicator in judging the performance of the resonance mode. It can directly reflect whether the resonance mode can be used in sensing applications. The higher the *Q* value, the better the sensing performance. According to the definition of the *Q* value, the *Q* value of the frequency point *D* can be up to 98.33, which is much larger than that of the frequency points *A* with *Q* of 6.46, *B* with *Q* of 13.62, and *C* with *Q* of 26.32. The large *Q* value of the frequency point *D* has potential applications in sensor-related fields. For the detailed discussion of it, please see below Fig. 5 and its text instructions.

**Fig. 3:**
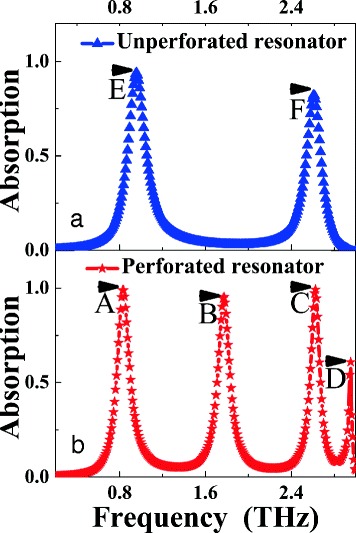
**a** and **b** are respectively the absorption performance of the unperforated and perforated rectangular light absorbers

To insight into the physical mechanism of the quad-band light absorber, we compare the absorption performance of the perforated rectangular resonator (i.e., the suggested structure in Fig. 1) and the unperforated rectangular resonator (i.e., without the air hole on the rectangular resonator), as shown in Fig. 3a, b. It should be noted that geometric parameters of those two kinds of absorbers are the same, except without the air hole for the unperforated rectangular resonator. For the absorption performance of unperforated rectangular resonator in Fig. 3a, two clear absorption peaks (marked as modes *E* and *F*) are achieved, the absorption rates of the two frequency points are 93.95 and 82.08%, respectively. By comparing the absorption performance of the Fig. 3a, b, we observed that the first (*A*) and the third (*C*) frequency points of the quad-band light absorber in Fig. 3b are very close to the frequency points *E* and *F* of the unperforated rectangular resonator in Fig. 3a. These characteristics show that the absorption mechanisms of the frequency points *A* and *C* of the perforated rectangular resonator should be respectively consistent to the mechanisms of the frequency points *E* and *F* of the unperforated rectangular resonator. The slight frequency differences should be due to the introduction of the air hole in the rectangular resonator.

**Fig. 4:**
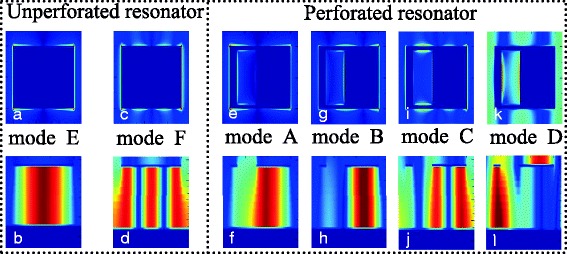
**a** and **c** show the |*E|* field distributions of the frequency points *E* and *F* of the unperforated rectangular resonator, respectively. **b** and **d** provide the |*H*y*|* field distributions of the frequency points *E* and *F* of the unperforated rectangular resonator, respectively. **e**, **g**, **i**, and (**k**) show the |*E|* field distributions of the frequency points *A*, *B*, *C*, and *D* of the perforated rectangular resonator, respectively. **f**, **h**, **j**, and **l** give the |*H*y*|* field distributions of the frequency points *A*, *B*, *C*, and *D* of the perforated rectangular resonator, respectively

To reveal the absorption mechanism of the frequency points *E* and *F* of the unperforated rectangular resonator, we give the electric (|*E*|) and magnetic (|*H*y|) field distributions of the two points *E* and *F*, as shown in Fig. 4a–d. It can be seen from Fig. 4b, d that the |*H*y| field distributions of the frequency points *E* and *F* are both primarily concentrated on the lossy dielectric layer. These distribution features show that the frequency points *E* and *F* are the localized responses of the unperforated rectangular resonator. The distribution of the magnetic field in the dielectric layer can lead to the accumulation of charge (or electric field) in the edges of the unperforated rectangular resonator [28, 39]. The |*E*| fields shown in Fig. 4a, c clearly demonstrate the excitation of electric field in the edges of the unperforated rectangular resonator. Furthermore, for frequency point *E* in Fig. 4b, there is only one strong field accumulation area in the lossy dielectric layer, which means that the frequency point *E* is the first-order localized resonance of the unperforated rectangular resonator [40, 41]. Different from the case of Fig. 4b, three field accumulation areas are observed for frequency point *F* in Fig. 4d. As a result, the frequency point *F* should be the third-order localized response of the unperforated rectangular resonator [40–43]. As mentioned at the end of the previous paragraph, the mechanism of the frequency points *A* and *C* in the perforated rectangular resonator should be the same as that of the frequency points *E* and *F* in the unperforated rectangular resonator, respectively. Therefore, we have reason to believe that the frequency points *A* and *C* should be respectively the first-order and third-order responses of the perforated rectangular resonator. In order to provide sufficient evidence, we need to analyze their field distributions.

We now provide the near-field distributions of the frequency points *A*, *B*, *C*, and *D* of the perforated rectangular resonator to reveal the physical mechanisms of the quad-band light absorber, as shown in Fig. 4e–l. It can be seen from Fig. 4f of the frequency point *A* that there is only one strong magnetic field distribution area in the dielectric layer of the suggested quad-band absorber device. Three accumulation areas (two strong and a weak) in Fig. 4j are found in the lossy dielectric layer of the quad-band absorption device for frequency point *C*. Meanwhile, the |*E*| field distributions of frequency points *A* in Fig. 4e and *C* in Fig. 4i are both mainly focused on the edges of the perforated rectangular resonator. Therefore, the frequency points *A* and *C* in Fig. 2a or Fig. 3b should be the first-order and third-order localized responses of the perforated rectangular resonator, respectively [40, 41]. These field distributions provide the sufficient evidence to show that the physical mechanisms of the frequency points *A* and *C* in Figs. 2a or 3b are consistent with the frequency points *E* and *F* in Fig. 3a, respectively.

For frequency point *B* in Fig. 4h, only one very strong field distribution is observed in the right side of the lossy dielectric layer and the |*E*| field of the absorption mode in Fig. 4g is mainly gathered in both edges of the right side section of the perforated rectangular resonator. As a result, the frequency point *B* should be the first-order localized response of the right side section of the perforated rectangular resonator. For frequency point *D*, we observed that its |*Hy*| field distribution is mainly focused on the left side of the lossy dielectric layer (see Fig. 4l), indicating this mode should be attributed to the first-order localized response of the left side section of the perforated rectangular resonator. Based on the above analysis, the introduction of the air hole on the rectangular resonator can indeed play a important role in the redistribution of the near-field patterns. The redistributed near-field distributions come into being two new absorption modes, the frequency points *B* and *D*. As a result, quad-band light absorption device can be realized in this slightly deformed rectangular resonator. Compared to the traditional design methods to obtain the multiple-band absorption [22–38], the concept of the design possesses obvious advantages, such as simplified structure design, low-cost and easy fabrication steps, and so on.

In this manuscript, we utilize the concept of the first-order and third-order resonances of the resonator to obtain the multiple-band absorption response. Generally speaking, however, any resonator possesses high-order modes in addition to the first-order response (or fundamental mode resonance), so from the theory, it can exhibit several absorption bands with high absorption coefficients within a certain frequency range. If the simulated frequency ranges are extended to the higher frequencies, one can find the other high-order modes, and the number of absorption bands with high absorption coefficients should be ideally infinite. However, the actual situation is not based on this prediction. Even if the frequency ranges are extended to higher frequencies, it is quite difficult to get a lot of (and even infinite) resonance bands having the high absorption coefficients, and typically only a maximum of two high-order resonance modes and a fundamental mode resonance can be achieved [40, 41, 47–49]. Two kinds of reasons can account for this phenomenon. Firstly, it is difficult to simultaneously achieve nearly perfect absorption at multiple different frequency bands (> 3) due to different optimal dielectric thicknesses required for the different resonance modes. In other words, it is impossible to realize the excellent absorption (simultaneous > 90%) of the multiple-band peaks based on the superposition of the fundamental mode and several (even infinite) high-order modes [40, 41, 46–48]. Secondly, the diffraction effects of the resonance structure can also considerably affect the absorption coefficients of the resonance peaks in fundamental mode and high-order responses and thus specific numerical investigation of the high-order modes to ensure that diffraction effects do not significantly influence their absorption performance [47–49]. The two points are the main reasons for not being able to obtain infinite nearly perfect absorption peaks even though the frequency ranges are extended to higher frequencies. Additionally, it is important to note that it is extremely difficult to obtain the even-order resonant modes under the normally condition (such as the vertically irradiated electromagnetic waves) because the electric field of the incident light must possess vertical components in the plane of incidence [49].

To give an intuitive demonstration, the dependence of the absorption spectra on the extended frequency ranges of the resonance device is provided in Fig. 2b. As shown, there are only four clear resonance modes (i.e., the original frequency points *A*, *B*, *C*, and *D*) with high absorption coefficients when the frequency ranges are extended to 4 THz, to 6 THz, and even to 8 THz. In the frequency ranges of (3~6) THz and (3~8) THz, some low absorption rates and unpredictable resonance modes can be found. This kind of feature indicates that we cannot obtain more resonance modes with high absorption coefficients and the expected frequencies when the frequency ranges are extended to higher frequencies. That is to say, the number of absorption bands cannot increase further (and even ideally infinite) with high absorption coefficients when the frequency ranges are extended to higher frequencies, which can be attributed to two reasons for the preceding paragraph.

Furthermore, we found that the absorption coefficients of these frequency points can be significantly affected when the frequency ranges are extended to higher frequencies. It can be seen from the curves of dark yellow, blue, and red of the Fig. 2b that the absorption coefficients of the first three frequency points are significantly decreased with the extending of the frequency ranges. Particularly, when the frequency range is extended to 8 THz, the absorption of the second frequency point is 67.69%; at the same time, the average absorption of the first three frequency points *A*, *B*, and *C* is only about 77.56%, which is much lower than the nearly perfect (or 100%) absorption of the first three frequency points in frequency range of the original (0.2~3) THz. Therefore, in this manuscript, we only discuss the resonance peaks (i.e., the modes *A*, *B*, *C*, and *D*) with high absorption coefficients of the frequency range of (0.2~3) THz without considering the cases of the low absorption coefficients and the unpredictable frequencies of the modes in the frequency ranges of (3~6) THz and (3~8) THz.

**Fig. 5:**
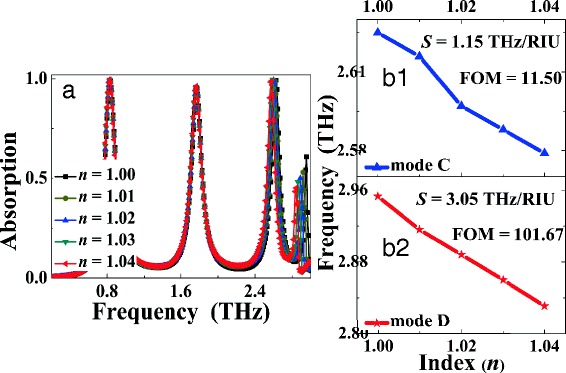
**a** shows the dependence of the absorption performance of the quad-band light absorber on the change of the refractive index (*n*) of surroundings. **b**_**1**_ and **b**_**2**_ are respectively the resonance frequencies of the modes *C* and *D* as the function of the refractive index *n*

We next investigate whether the designed quad-band light absorber can be incorporated into sensor to detect or monitor the change of the refractive index (RI) of surroundings, which are covered above the metallic resonator. Figure 5a shows the dependence of the absorption spectra on the change of the RI of the cover materials. It can be seen that the frequency shifts of the frequency points *A* and *B* are nearly absent (only 0.01 THz) when the RI is changed from vacuum *n* = 1.00 to *n* = 1.04 in intervals of 0.01, while frequency changes of the frequency points *C* and *D* are quite remarkable. The frequency change of the frequency point *C* is about 0.046 THz, and the shift of the frequency for the frequency point *D* can be up to 0.122 THz. In fact, the bulk refraction index sensitivity (*S*) is an intuitive factor to describe sensing performance of the resonance structure, and the sensitivity *S* can be defined as [44, 45]: *S* = Δ*f*/Δ*n*, where Δ*f* is the change of the resonance frequency and Δ*n* is the change of the RI. According to the definition, the *S* values of the frequency points *A*, *B*, *C*, and *D* are respectively 0.25, 0.25, 1.15, and 3.05 THz/RIU. Compared with the *S* values of the frequency points *A*, *B*, and *C*, the *S* enhancement factors for the frequency point *D* can be as high as 12.2, 12.2, and 2.65, respectively. The large *S* value of the frequency point *D* has potential applications in sensor-related areas.

Besides the sensing sensitivity *S*, the FOM (figure of merit) is a more significant factor to estimate the sensor quality and allows a direct comparison of sensing performance among different sensors. The definition of the FOM is [44, 45]: FOM = Δ*f*/(Δ*n ×* FWHM) *= S/*FWHM, where *S* and FWHM are the sensing sensitivity and the full width at half maximum of the resonance mode, respectively. Based on the *S* values and the FWHM of the four resonance modes, the FOMs of the frequency points *A*, *B*, *C*, and *D* are 1.92, 1.92, 11.5, and 101.67, respectively. The FOM of the frequency point *D* is about 52.95, 52.95, and 8.84 times larger than that of the frequency points *A*, *B*, and *C*, respectively. More importantly, the FOM of the frequency point *D* is much larger than previous works operated at the terahertz frequency range having the values of not exceeding 5 [18, 48–51]. Due to these excellent characteristics, the design of the multiple-band light absorber is promising in sensor-related fields.

## Conclusions

In conclusion, single-sized quad-band terahertz metamaterial absorber is demonstrated, which is designed by a perforated rectangular resonator on a lossy dielectric layer placed on a gold board. Four discrete and narrow-band resonance bands are achieved in the single-sized resonator, of which the first three bands have the large average absorption rates of 97.80% and the fourth band has high *Q* value of 98.33. The physical pictures of the designed device are explored; it is found that the corresponding near-field distributions of the four bands are different. Moreover, the dependence of the absorption on the refraction index change of the surroundings (which are covered above the single-sized resonator) is investigated to explore the device sensing performance. The FOM of the fourth band can reach 101.67, which is much larger than that of the first three modes and even previous works [18, 50–53]. These superior features, including high *Q* value and large FOM, will be beneficial to the design and development of simple sensors for gas sensing and monitoring, material detection, and bio-medical diagnostics.

## Abbreviations

EM: Electromagnetic
FOM: Figure of merit
*Q*: Quality factor
*S*: Sensing sensitivity
